# Enhancement of electrical characteristics and stability of self-patterned In–Zn–O thin-film transistors based on photosensitive precursors

**DOI:** 10.1038/s41598-020-76080-8

**Published:** 2020-11-02

**Authors:** Hee Jun Kim, Joohye Jung, Hyun Jae Kim

**Affiliations:** 1 School of Electrical and Electronic Engineering, Yonsei University, 50 Yonsei-ro, Seodaemun-gu, Seoul, 03722 Republic of Korea; 2 Display R&D Center, Samsung Display Co., Ltd, 181 Samsung-ro, Tangjeong-myeon, Asan-si, Chungcheongnam-do 31454 Republic of Korea

**Keywords:** Electronic devices, Electrical and electronic engineering

## Abstract

We report a novel self-patterning method for solution-processed indium zinc oxide (IZO) thin films based on photosensitive precursors. This approach is an alternative and evolutionary approach to the traditional photoresist patterning techniques. Chelate bonds between metal ions and β-diketone compounds in ultraviolet light-exposed IZO solutions provided intrinsic photosensitivity, which resulted in a solubility difference between exposed and non-exposed regions. This difference enabled self-patterning of the IZO for thin-film transistor (TFT) fabrication. Compared with previously reported self-patterning methods based on photosensitive activators, our self-patterned IZO TFTs based on photosensitive precursors displayed excellent electrical characteristics and stability. The field-effect mobility increased from 0.27 to 0.99 cm^2^/Vs, the subthreshold swing decreased from 0.54 to 0.46 V/dec, and the threshold voltage shift under a positive bias stress test (1,000 s) improved from 9.32 to 1.68 V. The photosensitive precursor played a key role in these improvements permitting fewer organic species which act as defect sites after metal oxide formation. Consequently, our approach compares favorably with that of conventional fabrication process using photoresist in terms of its simplicity, cost efficiency, and electrical performance.

## Introduction

Solution-processed electronic devices are of increasing interest due to their potential lower cost and vacuum-free fabrication processes. Unlike traditional semiconductor processing, low-temperature solution processes enable the deposition of metal oxides on a broader range of substrate materials (e.g., polyimide) and provide opportunities to produce flexible devices^1–3^. Since the first report of InGaZnO-based thin-film transistors (TFTs), solution-processed amorphous metal oxides have been regarded as some of the most promising materials for next-generation displays because of their high mobility and transparency^4–6^.

Meeting the needs of the times, numbers of solution-based processes have been developed, however, there remain barriers to commercialization, such as the patterning method^7^. Most applications require metal oxides to be finely patterned to form specific active areas in devices such as TFTs, solar cells, and analog circuits^8–11^.

Conventional photolithography process comprises many complex steps, including photoresist coating, vacuum drying, development, etching, and strip. This complexity limits not only the design of circuits, such as the pattern density, pattern profile, and width, but also the characteristics of processes, such as the thickness and profile of the photoresist, and chemical or physical damage to the etching^12^. Therefore, various alternative patterning methods have been developed such as micro-stamping and ink-jet printing^13–15^. Among them, a self-patternable solution process is of considerable interest because of its simplicity and low cost which means no specialized equipment is necessary. For instance, self-patterning of metal oxide using selective surface wetting has been reported. However additional layer was needed for surface modification; thus, plasma treatment was required to subsequently remove this layer^16^.

In previous study, we have suggested photoresist-free solution-processed amorphous oxide semiconductor TFTs using photosensitive sol-gels and determined the mechanism of the self-patterning process^17^. Photosensitive activators based on β-diketone compounds, such as benzoylacetone or acetylacetone (AcAc), play an important role in the presented self-patterning method. The addition of photosensitive activators to metal salts results in the formation of chelate bonds that lead to the crosslinking under ultraviolet (UV) light exposure allowing self-patterning of the metal-oxide film free of the photolithography process.

These results indicate the potential of the self-patterning method. However, some issues remain to be resolved, such as the reducing organic residues. Unwanted organic species from photosensitive activators and metal precursor ligands (e.g., nitrate (NO_3_^–^), chloride (Cl^–^), or acetate (CH_3_COO^–^)) remain after film formation; these species can cause degradation of TFT electrical characteristics and reliability^18^.

Herein, we report a new self-patterning method for solution-processed In–Zn–O (IZO) TFTs that focuses on the reduction of organic residues in the film. This was achieved using photosensitive precursors, rather than photosensitive activators^19^. The chemical and structural compositions of the self-patterned IZO thin films, based on the AcAc precursor and AcAc activators, were analyzed under the same conditions for comparison. Self-patterned IZO TFTs based on a precursor were named intrinsic photosensitive (IP) IZO TFTs, while self-patterned IZO TFTs based on an activator were named extrinsic photosensitive (EP) IZO TFTs. We confirmed enhancement of the electrical characteristics and stability of the IP IZO TFTs. The ease of processing, as well as the improved electrical characteristics and reliability of the TFTs, are expected to facilitate commercial realization of the self-patterning method.

## Experimental Section

### Preparation of the IZO solutions

For solution-processed oxide thin-film, synthesis of solution should be undertaken first. Indium acetylacetonate (In(OCCH_3_CHOCCH_3_)_3_) and zinc acetylacetonate hydrate (Zn(C_5_H_7_O_2_)_2_·xH_2_O) were selected as precursors to prepare the IP IZO solutions (0.3 M). The precursors were dissolved in 2-methoxyethanol (2ME) at various In:Zn molar ratio (i.e., 1:4, 1:3, 1:2, 1:1, and 2:1) to compare the patternability and electrical characteristics of the IP IZO thin films. An EP IZO solution (0.3 M) was prepared as follows. Indium nitrate hydrate (In(NO_3_)_3_·xH_2_O) and zinc nitrate hydrate (Zn(NO_3_)_2_·xH_2_O) were dissolved in 2ME at the In:Zn molar ratio of 1:2, which was the optimal ratio in terms of TFT electrical characteristics. This is the preparation method for conventional IZO solutions. Then, AcAc was added as photosensitizer. The mixture was stirred for 1 h at 70 °C and aged another 24 h at room temperature.

### Fabrication of IZO TFTs using the self-patterning method

**Figure 1:**
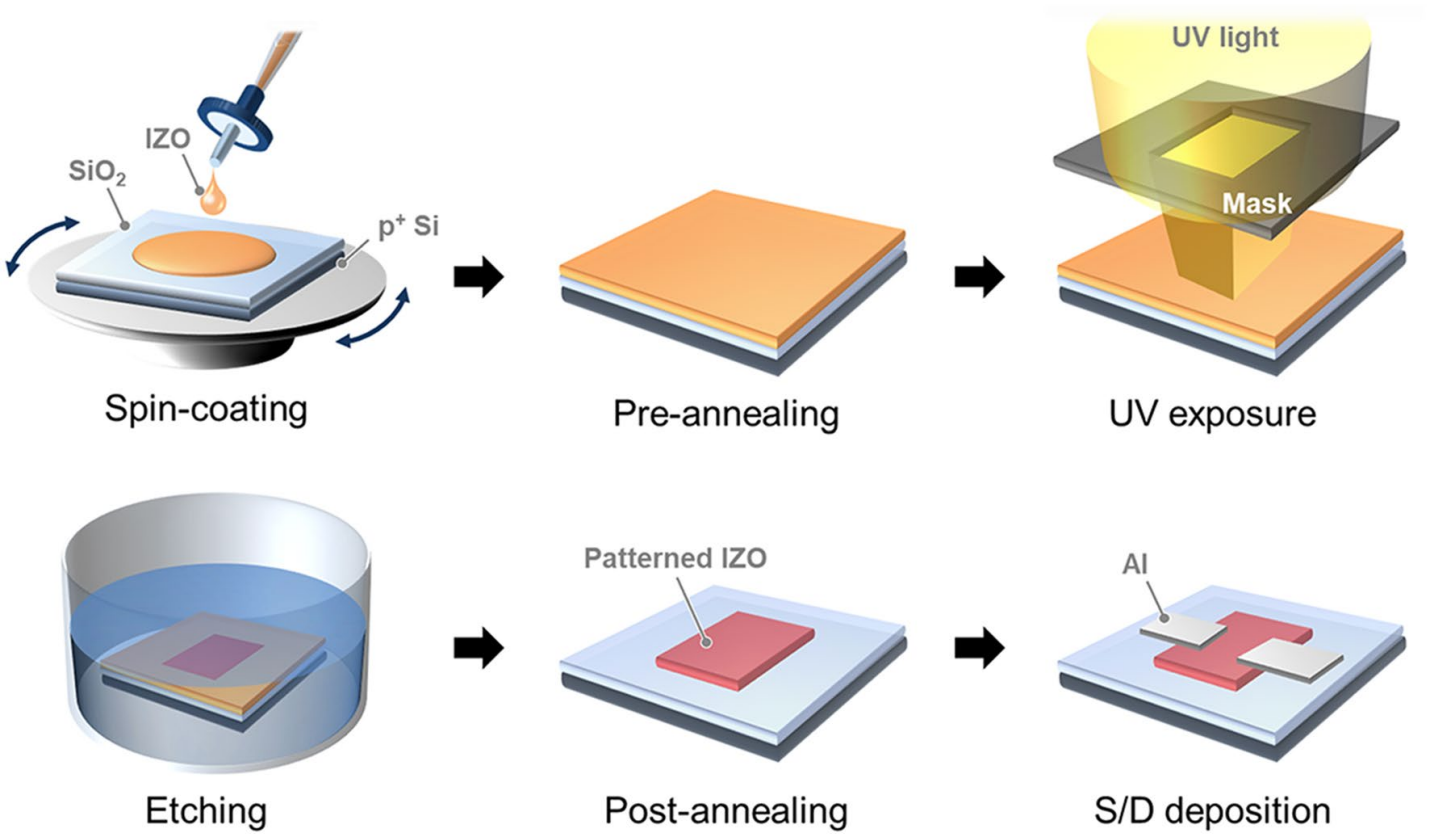
Schematic illustration of the experimental procedure.

The IP and EP IZO TFTs were fabricated under exactly the same conditions for comparison (Fig. 1). The device substrate was a heavily-doped p-type Si wafer on which a 1,200-Å-thick SiO_2_ layer had been thermally grown. After a standard cleaning procedure, the EP or IP IZO solution was spin-coated onto the substrate at 3,000 rpm for 30 s, then pre-annealed at 90 °C for 10 min. The pre-annealed IZO thin films were subsequently exposed to UV light—main emission peaks were at 253.7, 294.7, 302.2, and 365.0 nm—while a shadow mask was applied to the film for 15 min in ambient air. Following UV irradiation, the non-exposed IZO was etched by immersion in ethanol for 5 s, and post-annealed at 350 °C for 1 h in air. Source and drain electrodes were deposited by Al thermal evaporation; channel length and width are 150 and 1,000 μm, respectively.

### Film and device characterization

The optical absorbances of the IZO solutions at various wavelengths were measured using a UV–visible spectrophotometer (model V-650; JASCO). Images of the self-patterned IZO films were acquired using an optical microscope (model BX-51; Olympus). The chemical properties of the IZO thin films were analyzed by X-ray photoelectron spectroscopy (XPS) (model K-alpha; Thermo Fisher Scientific) and Fourier transform-infrared spectroscopy (FT-IR) (model Vertex 70; Bruker).

A Hall effect measurement system (model HMS 3000; ISTECH) was used to measure the semiconductor characteristics. The thickness of a patterned film was measured by atomic force microscopy (AFM) (model NX10; Park). The electrical characteristics of an IZO TFT were measured using a semiconductor parameter analyzer (model HP 4156C; Agilent Technologies) in a dark box under ambient conditions. The transfer curve was measured at a fixed drain voltage (V_DS_) of 10.1 V as the gate voltage (V_GS_) was swept from –30 to 30 V. A positive bias stress (PBS) test was carried out at a V_GS_ of 20 V and V_DS_ of 10.1 V for 1,000 s.

## Discussion

**Figure 2:**
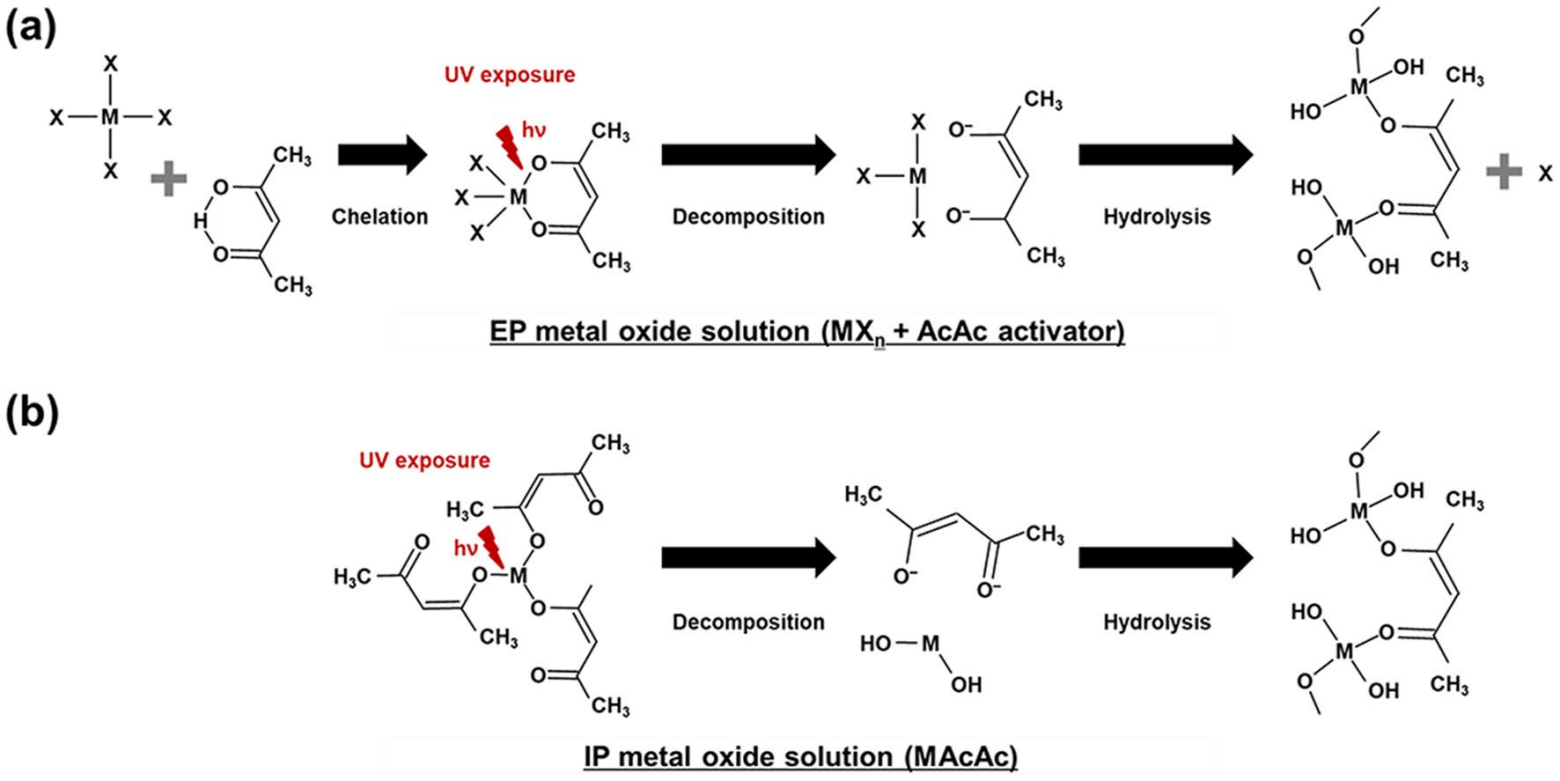
Schematic diagram of the crosslinking reactions of (**a**) EP and (**b**) IP metal oxide solutions according to UV irradiation.

In our previous research involving EP IZOs, metal salts (MX_n_; n = 4) dissolved in β-diketone compounds formed metal chelate bonds by exchanging the X ligands, such as chloride (Cl^–^), acetate (CH_10_COO^–^), or nitrate (NO_3_^–^). UV irradiation induced π → π* electronic transitions and decomposition of these chelate ring structures, which consisted of a central metal ion surrounded by β-diketonato ligands. The partial distribution of excited electrons on the oxygen atoms reduced the coordination strength between the metal ion and the β-diketonato ligands. As a result, this gradual decomposition allowed photosensitive behavior altering its solubility in some organic agent leading to hydrolysis of the IZO thin films^20,21^. This series of reactions is depicted in Fig. 2a.

On the other hand, in our newly developed method, the IP IZO was made using AcAc-based precursors, which already exhibited chelate bonds between the metal ion and the β-diketonato ligands. In this instance, fewer organic residues originating from the ligands were expected to remain after hydrolysis and crosslinking (Fig. 2b). In both instances, the dissociation of the metal chelate bonds under UV exposure provided photosensitivity. The solubility difference between the UV-exposed and non-exposed regions enabled patterning of the IZO thin film.

**Figure 3:**
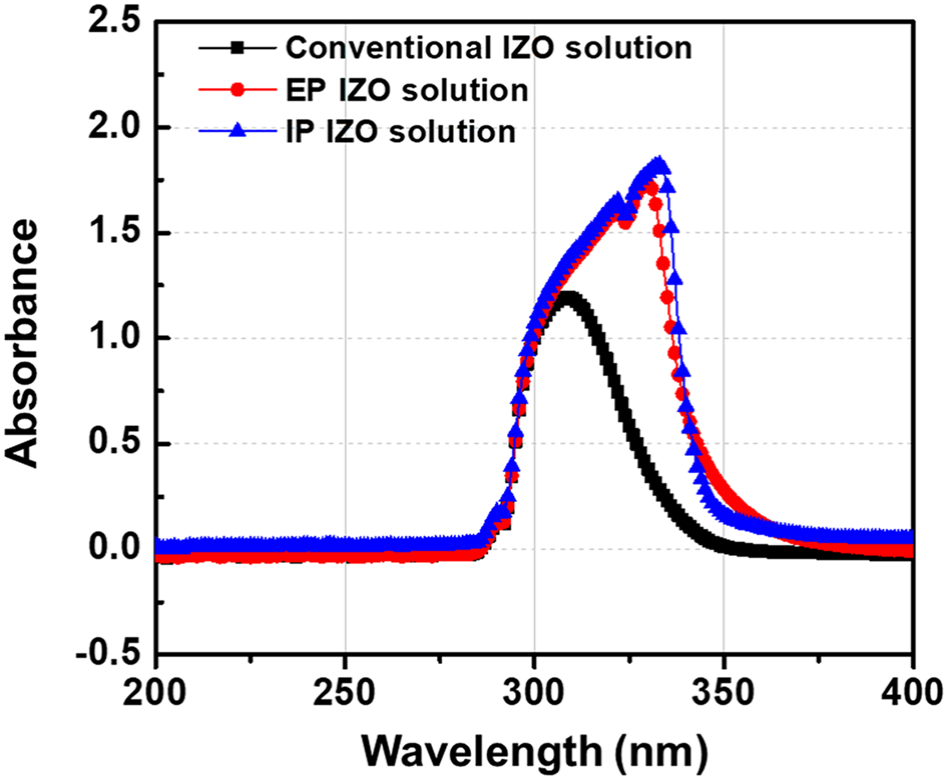
Optical absorption spectra of conventional, EP, and IP IZO solutions.

Figure 3 compares the optical absorption spectra of three types of IZO solutions: conventional, EP, and IP. The strong absorption bands of the IP and EP solutions between 300 and 350 nm originated from the metal chelate bonds. Regarding the EP IZO solution, the peak at 330 nm was attributed to the formation of metal chelate bonds between In^3+^ and Zn^2+^ metal cations and AcAc (In(AcAc)(NO_3_)_2_ and Zn(AcAc)NO_3_)^17^. A similar absorption peak at 330 nm observed for the IP IZO solution was attributed to the AcAc-based precursor.

**Figure 4:**
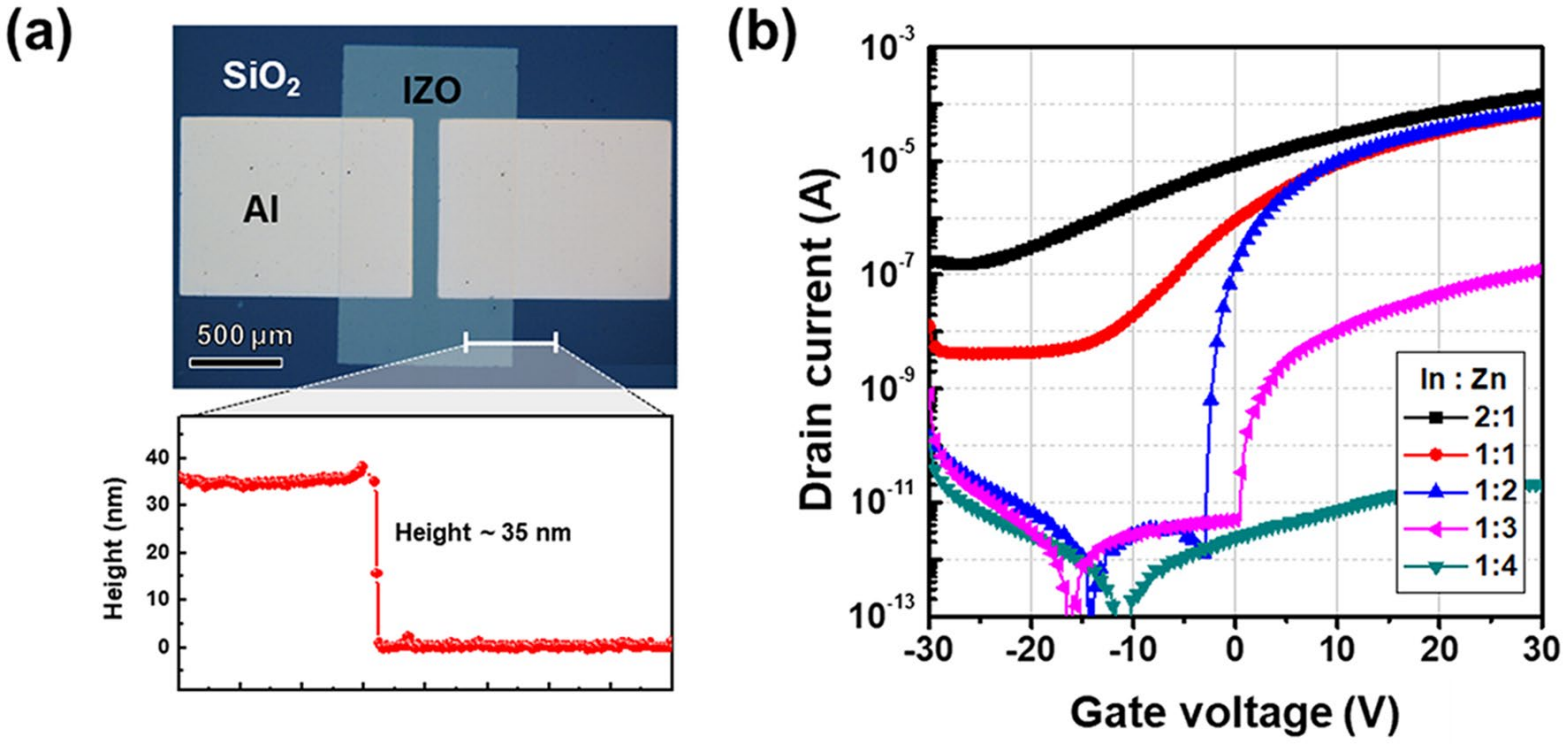
(**a**) Optical microscopy image and cross-sectional profile of IP IZO TFT. (**b**) Transfer characteristics of IP IZO TFTs with various In to Zn ratios from 2:1 to 1:4.

On the basis of above photoreactivity, we fabricated the IP IZO TFFs on the Si substrate as shown in Fig. 4a. A finely patterned 35-nm-thick IZO channel was made, which is suitable for TFTs with micron-sized lateral dimensions. Additionally, for practical applications, we demonstrated 3 inch wafer-scale patterned IP IZO films with various sizes and shapes (see Supplementary Fig. S1 online), and it was confirmed that the patterns such as elliptical and rectangular shapes were formed uniformly; the short axis of smallest ellipse is 40 μm and the width of the smallest rectangle is 60 μm. Figure 4b shows the transfer curves of IP IZO TFTs made with various In:Zn molar ratios (i.e., 1:4, 1:3, 1:2, 1:1, and 2:1). We previously established that In was a carrier supplier in the ZnO phase^22^. Herein, a similar trend was observed: elevated field-effect mobility (μ_FET_) from 0.01 to 1.55 cm^2^/Vs, reduced in I_on/off_ ratio from approximately 10^6^ to 10^2^, and increased subthreshold swing (S.S) from 0.93 to 12.49 V/dec with increasing In ratio. Table 1 summarizes the parameter variations. The clear switching behavior in the transfer curve for the In:Zn molar ratio of 1:2 indicated that this was the optimal ratio.

**Table 1:** Electrical parameters of IP IZO TFTs with various In to Zn ratios from 2:1 to 1:4.

In : Zn ratio	Mobility (cm^2^/Vs)	I_on/off_ ratio	S.S (V/dec)
2:1	1.55	9.54 × 10^2^	12.49
1:1	0.91	1.79 × 10^4^	5.23
1:2	0.99	1.00 × 10^9^	0.46
1:3	0.01	7.83 × 10^5^	0.93
1:4	Insulating (TFT always off)		

**Figure 5:**
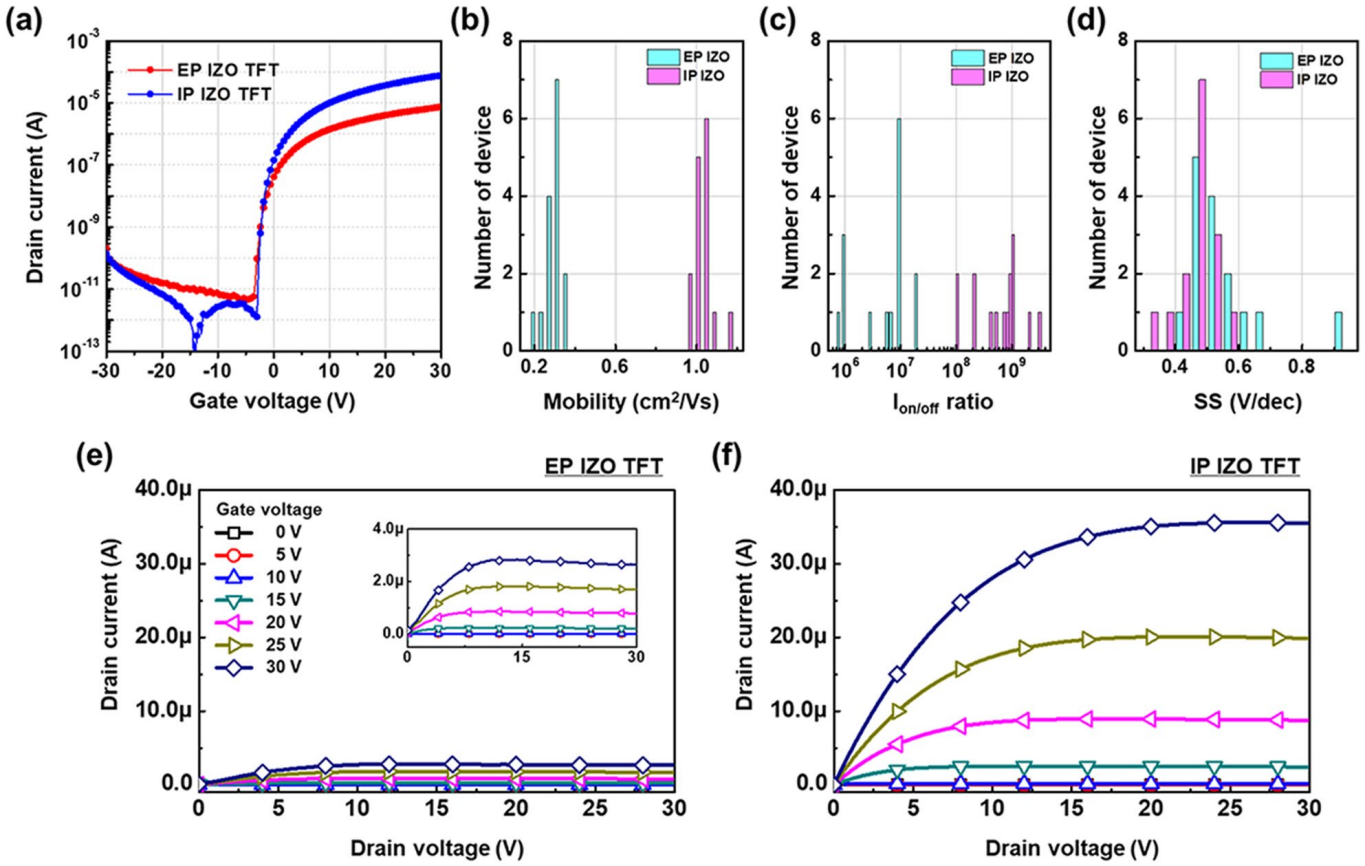
(**a**) Transfer characteristics of EP and IP IZO TFTs. Histograms of (**b**) field-effect mobility, (**c**) I_on/off_ ratio, and (**d**) S.S for EP and IP IZO TFTs (15 EA each). Output characteristics of (**e**) EP and (**f**) IP IZO TFTs.

Figure 5a shows the transfer curves of the IZO TFTs with the same molar ratio of 1:2, which were made from but with different types of EP and IP IZO solutions. Regarding the IP IZO, μ_FET_ of 0.99 cm^2^/Vs, I_on/off_ ratio of 1.00 × 10^9^, and S.S of 0.46 V/dec were observed. Meanwhile, the EP IZO exhibited relatively inferior characteristics with μ_FET_ of 0.27 cm^2^/Vs, I_on/off_ ratio of 1.07 × 10^7^, and S.S of 0.54 V/dec; the distributions of electrical parameters for the IP and EP IZO TFTs are summarized in Fig. 5b–d and Table 2. When comparing the output characteristics of EP and IP IZO TFTs in Fig. 5e,f, it was confirmed again that the current-level of IP IZO TFT is much higher than that of EP IZO TFT.

**Table 2:** Electrical parameters of EP and IP IZO TFTs (15 EA each).

Sample (15 EA)	Mobility (cm^2^/Vs)		I_on/off_ ratio		S.S (V/dec)	
	Mean	SD	Mean	SD	Mean	SD
EP IZO TFT	0.27	0.04	1.07 × 10^7^	0.87 × 10^7^	0.54	0.12
IP IZO TFT	0.99	0.05	1.00 × 10^9^	0.86 × 10^9^	0.46	0.06

SD, Standard deviation.

**Figure 6:**
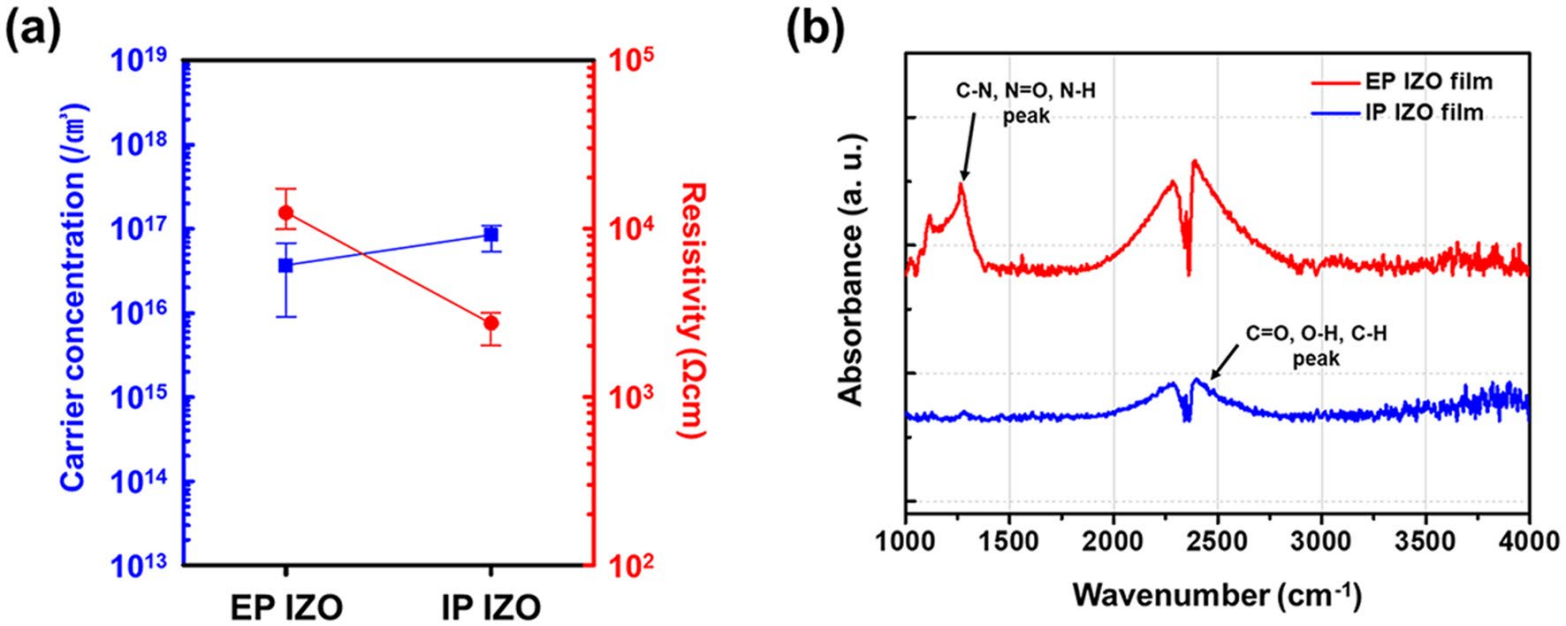
(**a**) Results of Hall measurement with error limitations (15 EA each) and (**b**) FT-IR spectra of EP and IP IZO thin films.

A similar trend was observed in the Hall measurement data (Fig. 6a). The IP IZO thin film showed elevated carrier concentration from 3.67 × 10^16^ to 8.43 × 10^16^/cm^3^ and reduced resistivity from 1.24 × 10^4^ to 2.73 × 10^3^ Ωcm, compared with the EP IZO thin film; these characteristics suggested fewer defects in the IP IZO phase. We previously reported that incomplete decomposition and volatilization of β-diketone photosensitive activators during metal oxide formation resulted in organics residues that degraded TFT performance. We presumed that these performance differences were derived from relatively low concentrations of residues in IP IZOs compared with the corresponding EP IZOs after film formation.

FT-IR spectroscopy of the IZO thin films was carried out to support this hypothesis (Fig. 6b). Peaks at 1,116 and 1,265 cm^−1^ in the EP IZO spectrum were assigned to nitrogen-related vibrations of molecules (e.g., C–N, N = O, and N–H) or as NO_3_^–^ deformations, most of which originated from nitrate precursors of EP IZOs^23–26^. Additionally, broad peaks in the range from 2,000 to 2,700 cm^−1^ indicated the presence of C=O, O–H, and C–H groups, which derived from incomplete formation of metal chelate bonds between metal ligands and AcAc^27,28^. These results confirmed the presence of considerable amounts of organic residues from the metal precursors and photosensitive activator that formed during film formation^29^. On the other hand, most peaks in the range from 1,000 to 1,500 cm^−1^ were absent in the spectrum of IP IZO. In the range from 2,000 to 2,700 cm^−1^, some residues from acetylacetonate metal precursors still remained, and relatively small absorbance peaks were shown as already predicted. These findings indicate that uniformly formed metal chelate bonds in acetylacetonate metal precursors are important for the quality of thin films.

**Figure 7:**
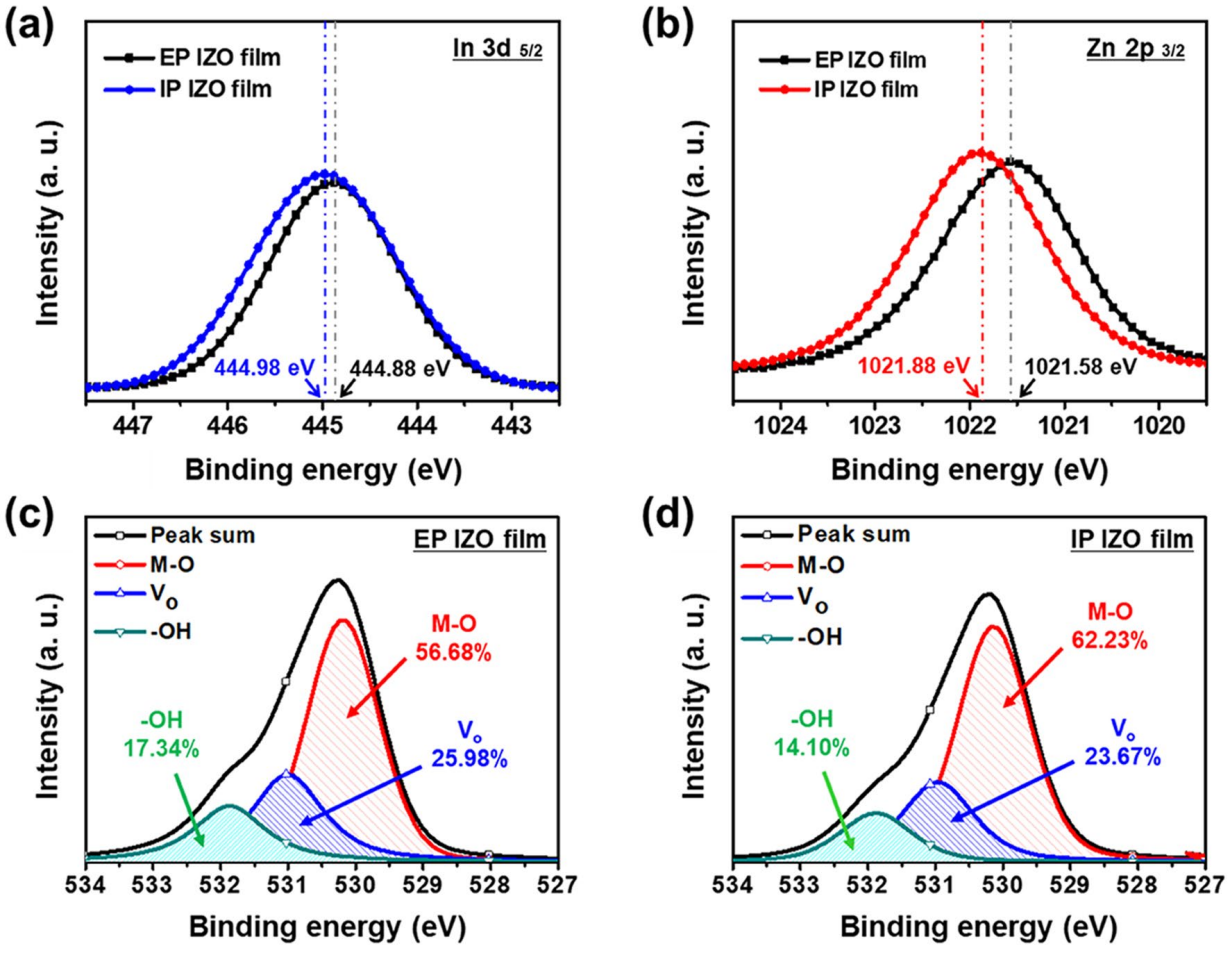
XPS analysis for (**a**) In 3d_5/2_ and (**b**) Zn 2p_3/2_ spectra of EP and IP IZO thin films, and O 1 s spectra of (**c**) EP and (**d**) IP IZO thin films.

Additionally, the chemical and structural compositions of the EP and IP IZO films were analyzed by XPS measurements. Figure 7a,b show the In 3d_5/2_ and Zn 2p_3/2_ peaks for the In–O and Zn–O bonds, respectively. For the IP IZO thin film, a shift toward higher binding energy from 444.88 to 444.98 eV and broadening of In 3d_5/2_ were observed which slightly broadened the peak compared with those of the EP IZO thin film. It has been reported that oxidized states of In-O compounds have higher binding energy and broader peak than In^30,31^. In this respect, the In 3d_5/2_ peak represent that more oxidation and better activation was occurred during IP IZO film formation. Same tendency was also shown in Zn 2p_3/2_ peak, slight shift to a higher binding energy, compared with those of the EP IZO thin film; this observation was consistent with the stronger tendency of the IP IZO thin film to undergo oxide formation^32,33^. Figure 7c,d show the O 1 s peak deconvoluted into three peaks centered at 530, 531, and 532 eV^34^.

The O_I_ peak at 530 eV, derived from the oxide lattice in the metal oxide phase, represents advanced conducting pathways for electrons in IP IZO (EP: 56.68%, IP: 62.23%). Meanwhile, the O_II_ peak at 531 eV is associated with oxygen vacancies (V_O_s), and the O_III_ peak at 532 eV is attributed to metal–hydroxide (M–OH) or metal–oxycarbon (M–OC) groups of organic residues^35^. The reduction in the intensity of these two peaks (i.e., O_II_ from 25.98% to 23.67%; O_III_ from 17.34% to 14.10%) indicated an overall reduction of oxygen impurities that generate trap sites and deteriorate performance. It has been reported that carbon-related species in IZO tend to strongly attract oxygen compared with In or Zn, and interfere with metal oxide (M–O) bond formation^36^. These reactions result in the formation of V_O_s (as evidenced by the O_II_ peak) and defect states. In this term, the smaller O_II_ peak for IP IZO indicated fewer defect states and better M–O bonding.

**Figure 8:**
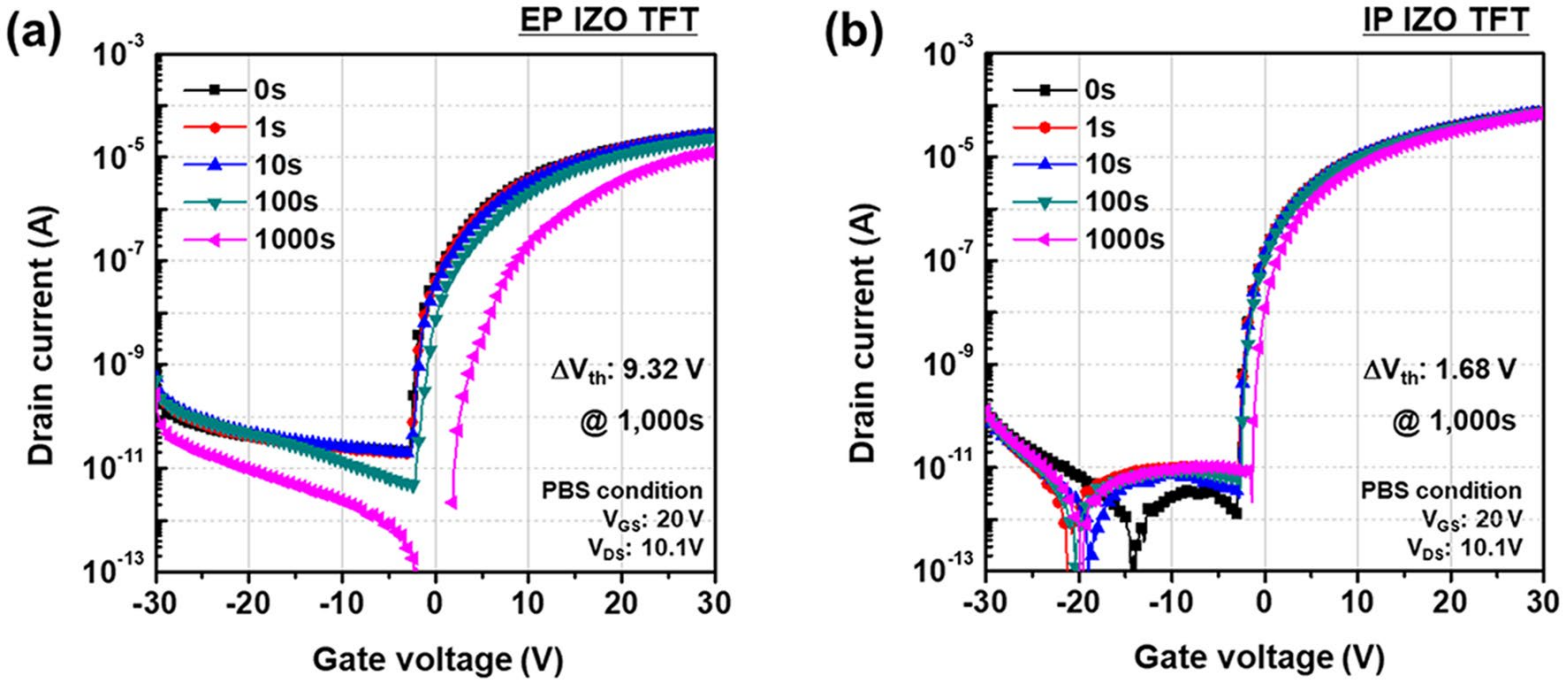
PBS test results of (**a**) EP and (**b**) IP IZO TFTs.

In the IP IZO solution, the only sources of carbon and oxygen impurities were the 2ME solvent and AcAc-based metal precursors. However, in the EP IZO solution, the AcAc activator was an additional impurity source in addition to the 2ME solvent and metal nitrate precursors. Consequently, these results figure out that the IP IZO thin film had enriched M–O bonding and contained fewer organic residues such as carbon or hydroxyl groups compared with the EP IZO thin film. The effect of these impurities was more discernable under bias stress test. Figure 8a,b shows the results of PBS test of the EP and IP IZO TFTs. The bias test was carried out at V_GS_ = 20 V and V_DS_ = 10.1 V for 1,000 s in the dark. As a natural consequence, the IP IZO TFT showed enhanced stability with a threshold voltage shift (ΔV_th_) of 1.68 V, compared with 9.32 V for the EP IZO TFT.

## Conclusion

In summary, we have demonstrated a novel self-patterning method for low-temperature solution-processed IZO TFTs. The AcAc based precursors provided intrinsic photosensitivity to the IZO solution, which self-patterned under UV irradiation. Compared with the conventional self-patterning method using photosensitive activators, our method provided higher-quality IZO thin films with fewer organic residues by limiting the possible sources of organic species to the 2ME solvent and the AcAc based precursors. By arranging M–O bonding of IZO uniformly with decreased V_O_s, a significant increase in the electrical characteristics in the transfer curve was achieved: μ_FET_ increased from 0.27 to 0.99 cm^2^/Vs, and S.S decreased from 0.54 to 0.46 V/dec. Furthermore, the ΔV_th_ under PBS tests improved from 9.32 to 1.68 V. Consequently, our new self-patterning method of IZO thin films is a commercially viable technique that reduces manufacturing cost and process steps due to non-necessity of photoresist but also contribute to superior electrical characteristics without degradation caused by organic residues.

## Supplementary information

Supplementary Information
