# Supplementary material for: Enhancement of electrical characteristics and stability of self-patterned In–Zn–O thin-film transistors based on photosensitive precursors
Source: Sci Rep. 2020 Nov 2;10:18853. doi: 10.1038/s41598-020-76080-8 (PMC7606435; doi:10.1038/s41598-020-76080-8)
Supplement: Supplementary file 1 — Supplementary Information [file 41598_2020_76080_MOESM1_ESM.pdf]

Supplementary information

Enhancement of Electrical Characteristics and Stability of Self-patterned In-Zn-O Thin-film Transistors based on Photosensitive Precursors

*Hee Jun Kim[1§], Joohye Jung,[1,2§], and Hyun Jae Kim[1]**

§ The first and second authors contributed equally.

[1]*School of Electrical and Electronic Engineering, Yonsei University, 50 Yonsei-ro, Seodaemun-gu, Seoul 03722, Republic of Korea*

[2]*Display R&D Center, Samsung Display Co., Ltd, 181 Samsung-ro, Tangjeong-myeon, Asan-si, Chungcheongnam-do 31454, Republic of Korea*

*hjk3@yonsei.ac.kr

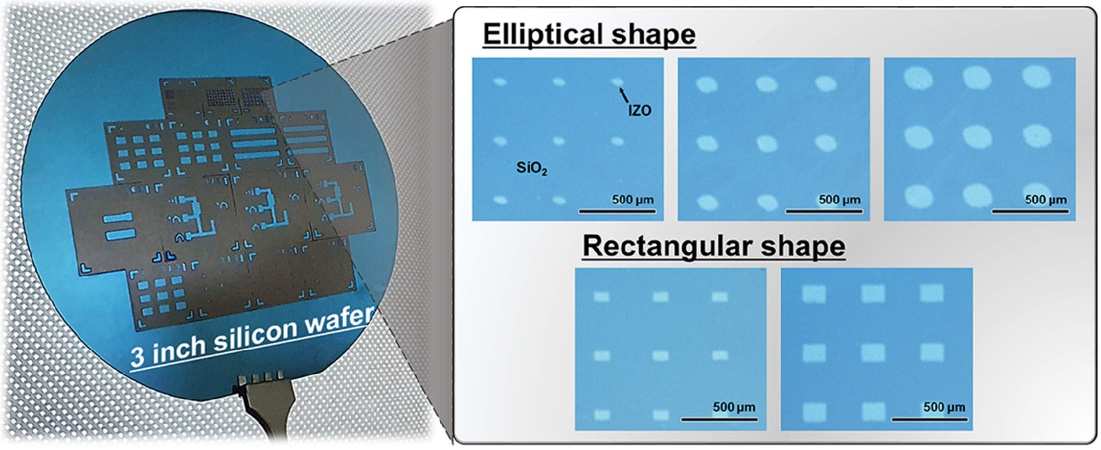

Figure S1. Demonstration of wafer-scale patterned IZO films and optical microscopy images of various patterns.

1. Supplementary experimental section

1.1. UV-visible spectroscopy

Three types of IZO solutions (conventional, EP, and IP) were prepared to measure the optical absorption spectra. For conventional IZO solution, indium nitrate hydrate ($In(NO_3)_3 \cdot xH_2O$) and zinc nitrate hydrate ($Zn(NO_3)_2 \cdot xH_2O$) were dissolved in 2ME. In the case of the EP IZO solution, acetylacetone (AcAc) was added to the conventional IZO solution as photosensitizer. For the IP IZO solution, indium acetylacetonate ($In(OCCH_3CHOCCH_3)_3$) and zinc acetylacetonate hydrate ($Zn(C_5H_7O_2)_2 \cdot xH_2O$) were selected as precursors. All solutions have the In:Zn molar ratio of 1:2 and the molar concentrations were fixed to 0.3 M. The mixtures were stirred for 1 h at 70°C and aged another 24 h at room temperature. The optical absorbances of the IZO solutions at various wavelengths were measured using a UV-visible spectrophotometer (model V-650; JASCO). To measure the optical absorbance of IZO solution, a cuvette was used (product code HRA-2810100; RATIOLAB), composed of polymethylmethacrylate (PMMA). The optical path of light passing through the cuvette is 10 mm.

1.2. Hall effect measurement

To measure the semiconductor characteristics of the EP and IP IZO films, the EP and IZO films were prepared on the glass substrates. The EP and IP IZO films were fabricated under exactly same conditions. After the cleaning procedure of the substrate, the EP or IP IZO solution was spin-coated onto the substrate at 3,000 rpm for 30 s, then pre-annealed at 90°C for 10 min. The pre-annealed IZO films were subsequently exposed to UV light for 15 min in ambient air. Following UV irradiation, the UV-exposed IZO films were post-annealed at 350°C for 1 h in air. The fabricated IZO films were cut into 5 mm × 5 mm size using the diamond cutter, and the silver paste was coated on each corners. The carrier concentration and resistivity of EP and IP IZO films were

measured in air, at room temperature, and under a magnetic field of 0.51 T using a Hall effect measurement system (model HMS 3000; ISTECH).
